# Anxiety and pain perception in patients undergoing mandibular autogenous block bone surgery

**DOI:** 10.4317/jced.55595

**Published:** 2020-02-01

**Authors:** Luiz-Felipe-Silva Novy, Evandro-Guimarães Aguiar, José-Alcides-Almeida de Arruda, Allyson-Nogueira Moreira, Emerson-Gomes dos Santos, Cláudia-Silami de Magalhães, Amália Moreno

**Affiliations:** 1 DDS, MSc, Department of Oral Surgery, Pathology and Clinical Dentistry, School of Dentistry, Universidade Federal de Minas Gerais, Belo Horizonte, Minas Gerais, Brazil; 2 DDS, PhD, Professor, Department of Oral Surgery, Pathology and Clinical Dentistry, School of Dentistry, Universidade Federal de Minas Gerais, Belo Horizonte, Minas Gerais, Brazil; 3 DDS, PhD, Professor, Department of Restorative Dentistry, School of Dentistry, Universidade Federal de Minas Gerais, Belo Horizonte, MG, Brazil; 4 BStat, PhD, Professor, Department of Business, Paulista School of Politics, Economics and Business, Universidade Federal de São Paulo, Osasco, SP, Brazil

## Abstract

**Background:**

The aim of the present study was to investigate pain perception and anxiety within the context of surgery for the placement of mandibular block bone and to evaluate the causality effect between theses variables.

**Material and Methods:**

A total of 13 patients were recruited for the study and were submitted to mandibular autogenous block bone surgery. Demographic data were collected and the anxiety level was determined using the State-Trait Anxiety Inventory (STAI). The STAI was administered on the day of surgery and on the 14th postoperative day. Pain was determined using the visual analogue scale (VAS) and limitation of daily activities and postoperative symptoms were also reported. Data were analyzed using parametric tests (α=0.05) and cross-lagged analysis was performed to verify a causality effect.

**Results:**

Few patients reported interference with daily activities or the presence of postoperative symptoms. A significant association of bad breath/taste with STAI-State was detected on the 14th postoperative day. No evidence of causality between STAI and VAS was detected.

**Conclusions:**

The patient’s self-evaluation indicates that the pain and anxiety level felt during treatment was not directly associated with the clinical aspects of the surgical procedure or with postoperative activities/symptoms limitations.

** Key words:**Anxiety pain, questionnaires, autogenous bone block, treatment, outcomes.

## Introduction

Oral surgical procedures are common for individuals requiring rehabilitation with dental implants or after bone grafts surgeries (1). Autogenous intraoral bone grafts have been widely employed to increase the bone volume of the alveolar crest due to their reliability and predictability (2). The use of autogenous block grafts for vertical and horizontal bone augmentation has been reported, with a low rate of complications and satisfactory bone regeneration at the donor site (3,4).

These procedures are known to involve mild to moderate pain, causing stress and short-term limitations of daily activities, especially during the first postoperative days (5,6). In addition, previous studies have demonstrated an impact of patient anxiety on factors related to dental implants surgery (7,8). The control of anxiety levels in patients submitted to oral and maxillofacial surgeries may be directly related to the subjective control of pain and its impact on daily activities, with a consequent reduction of morbidity in the operated region (9,10).

Dental anxiety has not been clearly defined in the literature, ranging from the mildest feeling of apprehension before a clinical procedure to the strongest panic attack with impairment of dental treatment (11). Gaudry *et al.* (12) described the difference between anxiety as a transitory state in the face of threatening stimuli and as a personality trait. State anxiety is based on subjective and conscious feelings of apprehension and tension accompanied by, or associated with, activation of the autonomic nervous system. In contrast, trait anxiety implies a motive or an acquired behavioral disposition that predisposes an individual to perceive as threatening circumstances that are not objectively dangerous and to react with state anxiety of disproportionate intensity related to the magnitude of danger (12).

The emotional status of an individual who undergoes dental surgery may generate or worsen a degree of anxiety that will have some type of impact on the treatment effectiveness. Although the effect of anxiety on patient pain after dental implant or extraction procedures has been investigated (6,13) to our knowledge, no reports on patients submitted to bone graft surgeries are currently available. Intraoral bone grafting may be affected by various factors such as duration of surgery, trans- and postoperative intercurrences, duration of pain, and recovery, which contribute to a state of stress. Thus, the aim of the present study was to evaluate the pain perception and anxiety related to autogenous mandibular block bone surgery using visual analog scales (VAS), the State-Trait Anxiety Inventory (STAI), and information about treatment and follow-up data. Also, we evaluated the causality effect of anxiety on pain perception during the treatment period.

## Material and Methods

-Study design and ethical issues

A prospective cohort study was conducted on individuals seen at the Service of Oral Surgery and Implantology of the School of Dentistry, Federal University of Minas Gerais (MG, Brazil) between July and December 2017. The study was approved by the local Ethics Committee (Approval No. 67497617.7.0000.5149). The participants gave written informed consent in agreement with the Declaration of Helsinki.

-Patients

A total of 13 individuals were included in the study according to the criteria: adult or elderly needing a thick bone graft in the posterior mandible as determined by visual examination and palpation and confirmed by cone beam computed tomography (CBCT); 3.0 mm to 8.0 mm bone height remaining above the mandibular canal roof; valley-shaped bone defect with a 2.0 mm minimum depth in relation to the bone crest, with indication of oral rehabilitation with bone integrable implants; and cognitive ability to understand and answer the questionnaires.

Exclusion criteria were as follows: general contraindications of surgical treatment, being under treatment or taking medications that interfere with tissue repair, smokers and alcoholics, presence of active infectious process in the region to be operated, and a history of implant or graft installation in the mandible.

Gender, age, household income, reason for the dentist’s appointment of the subjects were recorded. Intraoral physical examination was performed, and preoperative laboratory tests and CBCT images were evaluated. The responses to two self-applicable questionnaires were collected.

-Surgical procedures

All surgical procedures were performed by the same senior maxillofacial surgeon (E.G.A.). Two calibrated examiners (L.S.N. and C.S.M.) conducted the clinical examination and collected the VAS and questionnaire data.

The autogenous bone graft was obtained from the external oblique ridge according to Khoury *et al*. (4), with some modifications. The following preoperative medication was used: amoxicillin (875 mg, administered at least 1 hour prior to surgery), tenoxicam (20 mg, at least 1 hour prior to surgery), dexamethasone (two 4 mg capsules, at least 12 hours prior to surgery, and 8 mg at least 2 hours prior to surgery), and paracetamol (750 mg, at least 1 hour prior to surgery).

**Figure 1:**
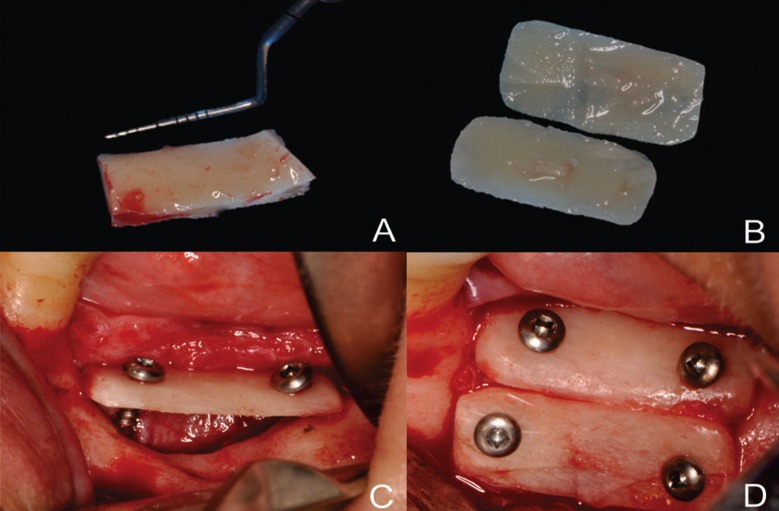
(A) Harvesting two bone blocks from the mandible, (B) splitting them into two thinner blocks, and (C,D) placement of bone blocks in occlusal and vestibular bone cortices.

Perforations were punched in the anesthetized donor area with an NR699 drill (Solidor Ind., SP, Brazil) in parallel to the external oblique area, reaching the full thickness of cortical bone. Mesial and distal vertical osteotomy lines were performed under constant irrigation with saline (5ºC), determining the length of the graft. The vertical osteotomies were then joined with a horizontal osteotomy at the basal level using a diamond disk (5 mm, NeoBiotech, CA, USA). Particulated bone from a region adjacent to the donor bed was obtained with the aid of an ACM drill (NeoBiotech, CA, USA). The bone block was carefully removed with a chisel and divided longitudinally in the middle into thin parts with a diamond disk (Fig. 1). The edges were rounded and the blocks were stabilized in the recipient regions at a distance from the bone bed and maintained with titanium screws (1.5 mm, NeoBiotech, CA, USA) (Fig. 1) manually inserted using a screwdriver key (square active tip, PecLab Ind., MG, Brazil). The first block was fixed in the occlusal region resting on the bone crest of the adjacent tooth. The space below the first block was filled with particulate bone and the second block was then installed on the vestibular surface.

The surgical wounds in the recipient and in the donor areas were then closed with simple non-restorable sutures (Nylon 6-0, Ethicon, NJ, USA). The number of anesthetic tubes used, the surgical time and transoperatory intercurrences were recorded.

-Follow-up and daily activities/postoperative symptoms

Medication to be continued postoperatively with amoxicillin (875 mg twice a day for 7 days), tenoxicam (20 mg for 5 days), dexamethasone (8 mg twice a day for 5 days), and paracetamol (750 mg four times/day for two days). After the third postoperative day, the patients were instructed to perform mouthwashes with 5 mL 0.12% chlorhexidine gluconate for 1-minute half an hour after oral hygiene, three times a day for 7 days.

All participants were evaluated seven days after surgery and the sutures were removed after 10 days, when clinical reevaluation was performed. The patients were instructed not to apply a direct load to the reconstructed region with the use of dental prostheses throughout the phase of bone regeneration. After the 14th postoperative day the patients were asked whether they experienced interference with their daily activities such as chewing, full mouth opening, speaking, sleeping, job, social life, or favorite activities. The patients were also asked to report postoperative symptoms such as the presence of facial swelling, nausea, and the perception of bad breath/taste.

-STAI

For the assessment of anxiety, the patients responded to two self-assessments questionnaires (14,15): STAI-State (STAI‑S), and STAI-Trace (STAI‑T), seven days before surgery (phase 1), immediately (30 minutes) before surgery (phase 2) and 14 days after surgery (phase 3). The state anxiety scale consists of 20 statements that assess how the respondent feels “at this time”. The trait anxiety scale consists of 20 statements that assess how the respondent “generally feels”. Both scales evaluate feelings of apprehension, tension, nervousness and worry. The patients expressed each statement using a 0 to 4 Likert-type scale.

-VAS

Each patient performed the VAS test, recording the perception of pain on a scale from 0 to 10, with 0 corresponding to “no pain” and 10 to “the worst pain ever felt”. The test was performed 30 minutes after bone graft surgery (phase 2), and again 14 days after surgery (phase 3).

-Statistical analysis

Demographic data and the reasons for seeking dental treatment were submitted to descriptive analyses using the Statistical Package for the Social Sciences software (SPSS), version 22.0 (IBM Inc., New Armonk, NJ, USA). The sum of STAI scores for all questions for each patient was calculated. Scores of 20-40 represented a low level of anxiety and scores of 41-80 represented an medium to high level of anxiety. The responses to the VAS were dichotomized as “without pain” for zero scores and “with pain” for scores from 1 to 10. The hypothesis of association between clinical variables and STAI was tested at phase 2; between daily activities/postoperative symptoms and VAS at phase 3, and STAI at phase 3. Fisher’s exact test of independence (α=0.05) was used to verify the hypothesis of association as the sample size is small.

STAI sums of scores were compared at 3 time points (phases 1,2 and 3) using the Friedman test. A post-hoc test (α=0.05) was employed using the PMCMR package of the R software (R Core Team, Vienna, Austria). The hypothesis of no association between the patients’ STAI and VAS were tested by cross-lagged analysis using the COCOR package (16) of the R software. Thus, three types of correlations were performed: autocorrelations (between two measures of a variable at different times), synchronous correlations (between different variables at the same time) and cross-lagged correlations (between different variables at different times). The difference between cross-lagged analyses was determined by the modified Pearson-Filon ZPF test.

## Results

The sociodemographic data of the individuals are shown in  Table 1. The sample consisted of nine women and four men (mean age: 53.4 years). Approximately 50% of the individuals had household incomes lower than 3 times the Brazilian minimum wage, and the other 50% had incomes higher than this level. The main reason for the dental appointment was the recovery of missing teeth (69.2%).

**Table 1:**
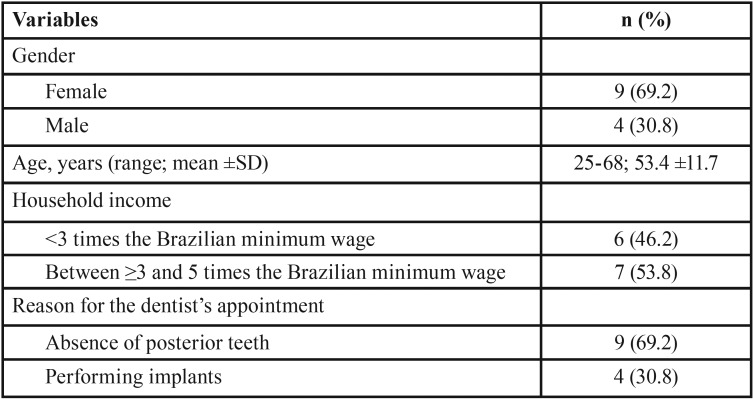
Sociodemographic data of participants undergoing bone surgery.

No significant association between clinical variables and STAI (*p*>0.05) was observed at phase 2 ( Table 2). Some factors such as duration of surgery (*p*=0.078), complications during surgery (*p*=0.070) and number of anesthetic cartridges used (*p*=0.098) may have been influenced statistically by anxiety considering an interval estimate with a 90% confidence level ( Table 2). Considering some influence of surgery duration on anxiety five (71.4%) subjects whose surgery lasted more than 120 minutes showed medium to hight anxiety on STAI-S at phase 2, in contrast to one (16.7%) patient whose surgery lasted less than 120 minutes (*p*=0.078). Only three patients showed complications during surgery, also showing average to high anxiety on STAI-S at phase 2 (*p*=0.078) ( Table 2).

**Table 2:**
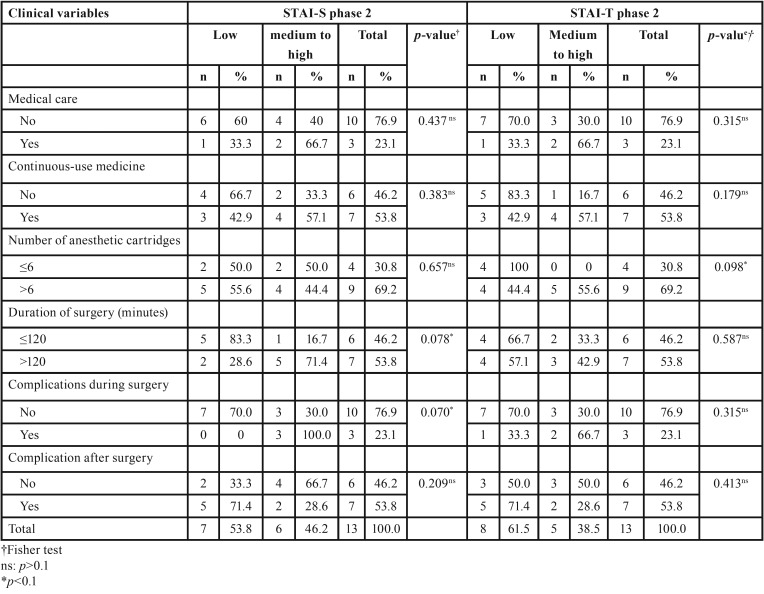
Association between State-Trait Anxiety Inventory (STAI) scores and clinical variables.

In addition, regarding daily activities/postoperative symptoms, an association was detected between bad breath/taste and STAI-S at phase 3 (*p*=0.014). Three of the four (30.8%) patients who reported bad breath/taste (75.0%) showed medium to high anxiety on STAI-S at phase 3 (*p*=0.014). There was no significant association between STAI at phase 2 and other variables (*p*>0.05) ( Table 3).

**Table 3:**
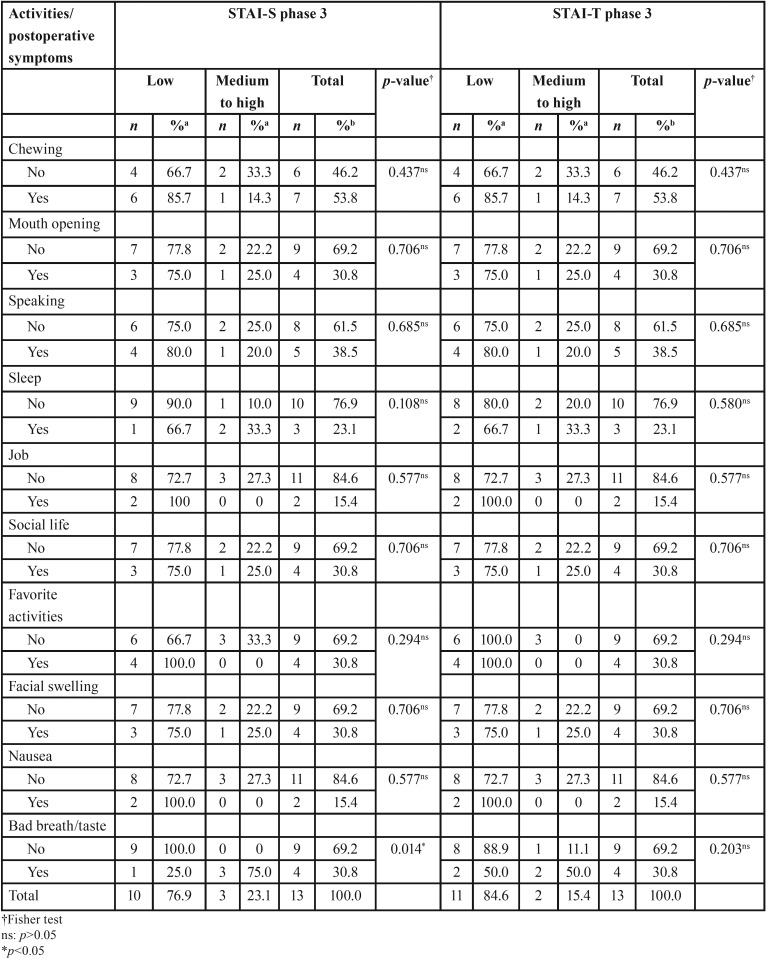
Association between State-Trait Anxiety Inventory (STAI) scores and daily activities/postoperative symptoms.

**Figure 2:**
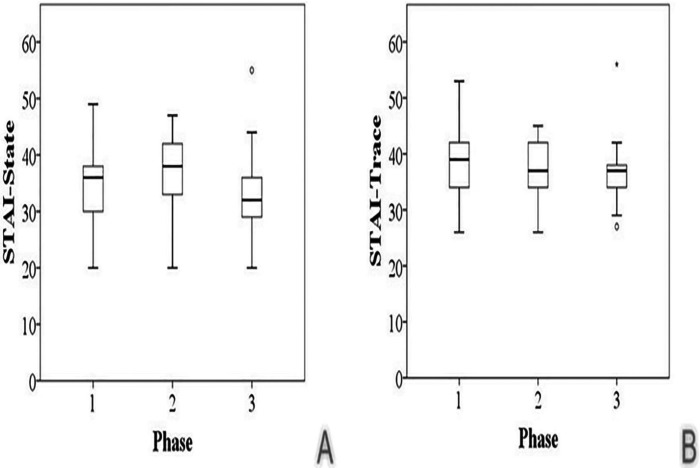
Box Plot of STAI-S (A) and STAI-T (B) at different phases of the study.

Regarding association between activities/symptoms and VAS at phase 2, four (30.8%) patients complained of interference with their favorite activities and two (50%) reported some level of pain ( Table 4). Also, there was no significant difference for STAI between the three phases of this study (*p*>0.05, Friedman test) (Fig. 2). Regarding VAS, six patients reported pain at phase 2 compared with two patients at phase 3, with a statistically significant difference between phases 2 and 3 (*p*=0.014, Wilcoxon test).

**Table 4:**
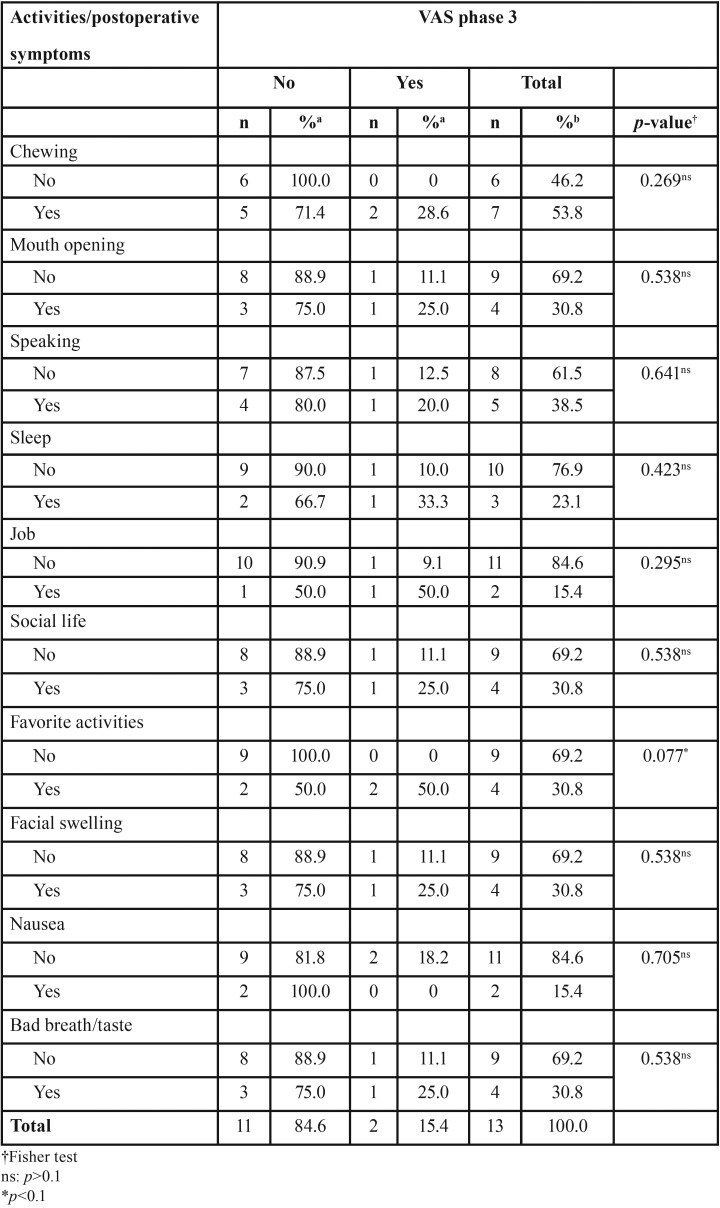
Association between visual analog scales (VAS) and daily activities/postoperative symptoms.

**Figure 3:**
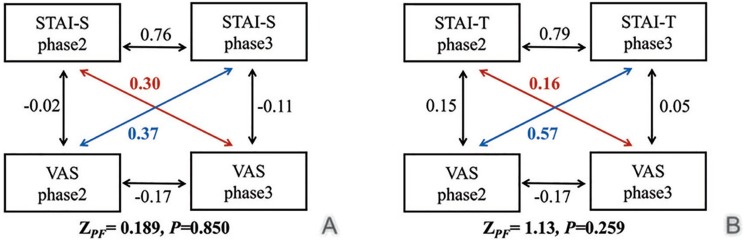
Cross-lagged analysis for STAI-S and VAS (A) and STAI-T and VAS (B).

The autocorrelations in the STAI responses between phases 2 and 3 were significant (r=0.76, *p*=0.003 to STAI-S; r=0.79, *p*=0.001 to STAI-T). There were no significant differences in synchronous correlations at phases 2 or 3 between STAI responses and VAS. Cross-lagged analysis showed significant causality only between VAS at responses phase 2 and STAI-T responses at phase 3 (r=0.57; *p*=0.044). In general, cross-lagged analysis of the data did not show significant causality between STAI and VAS responses (ZPF test, *p*>0.05) (Fig. 3).

## Discussion

Marked bone loss in the edentulous region of the posterior mandible requires surgical procedures in order to obtain vertical and horizontal bone gain before treatment with dental implants (2,17). Since surgical procedures are considered to be negative by involving pain, the present study revealed that 69.2% of the patients studied sought oral rehabilitation in order to restore the lost posterior teeth, whereas a minority of the sample seemed to already have been informed about surgery for dental implants and to be willing to undergo such treatment. Moreover, the emotional status of medium to high anxiety of same patients at phase 2 was associated with some factors inherent to the procedure such as surgical time, complications and number of anesthetic cartridges during the operation. This fact may be related to evidence that more anxious patients are usually restless and interfere with the progression of the surgical procedure (8,18).

Interference with routine activities (e.g., working or studying) has been reported during the first three postoperative days, followed by reduced pain along the first postoperative week (5). These findings agree with the results of the present study, since most patients submitted to bone graft surgery reported pain during the immediate postoperative period, with a significant difference compared to 14 days after surgery. However, this fact may be probably due to the comforTable condition provided by the surgical procedure. Also, the fact that most limitations of daily activities and the postoperative symptoms did not show an association with anxiety is due to the good oral and systemic conditions provided by graft surgery. Similar studies have reported that patients experience pain of mild to moderate intensity in surgeries for dental extraction or for surgical implants, usually showing a good recovery, including monitoring for future procedures (5,8,19). However, bad breath was observed in association with STAI-S. Our findings agree with the study of Muglali *et al.* (19), who reported that some underestimated factors related to oral health may be associated with the presence or absence of a reduction of patient anxiety before and after oral surgery.

Herein, there was no significant difference during the phases of evaluation of anxiety, a fact that supports the permanence of the emotional status and the expectation about the treatment received. Although cross-lagged analysis did not show a statistically significant difference in causality, it can be seen that the pain occurring in phase 2 may have influenced the anxiety during phase 3 in the STAI-T. The factors that may be involved in anxiety are the age of the subjects, their psychological conditions, previous medical experiences, different degrees of sensitivity to, or tolerance of, pain, influence of the family or the peer group regarding the potentiation or minimization of the personality status, as well as the reaction to the place of care (20). Previous studies have investigated the theme of origin of dental anxiety, its main causes and consequences related to the fear previously shown by the patient (21,22). In addition, dental fear and anxiety may have generalized psychosocial consequences, explaining the permanence or increase of anxiety during treatment (22).

A positive association between anxiety and the perception of pain in surgical dental procedures was reported (6,23). Moreover, the technical procedure and the surgical skill of the operator are important factors for the success of intraoral bone graft surgery, as the patient emotional control becomes a requisite for surgical success (23). Thus, it is important for the professionals to understand that a painful experience can influence anxiety and that the anxiety state is related to changes in the execution of future treatments.

The limitations of the present study are due to the aspects inherent to the design of a prospective study, so that the evaluations of the subjects must be systematically understood and controlled. Suggested perspectives for future investigations are longitudinal studies monitoring patients submitted to bone graft surgeries in order to perform a qualitative analysis of these individuals when facing these procedures.

## Conclusions

Within the limitations of the current study, we may conclude that some clinical variables related to surgical treatment and to the limitation of activities/postoperative symptoms appear to be associated with the level of anxiety during surgical treatment for autogenous bone block graft. The perception of pain immediately after surgery did not predict the STAI-S after treatment, and there was an indication of causality regarding the STAI-T.
